# The association between contact sport exposure and cervical sensorimotor dysfunction: a scoping review of implications for future musculoskeletal injury risk

**DOI:** 10.1186/s12998-022-00458-w

**Published:** 2022-11-24

**Authors:** Kelly Cheever, Jeffery King, Keisuke Kawata

**Affiliations:** 1 Applied Biomechanics Laboratory, Department of Kinesiology, College of Health, Community and Policy, University of Texas at San Antonio, One UTSA Cir, San Antonio, TX 78429 USA; 2 Department of Neurosurgery, Medical College of Wisconsin, Milwaukee, WI USA; 3 Department of Kinesiology, School of Public Health-Bloomington, Indiana University, Bloomington, IN USA; 4 Program in Neuroscience, College of Arts and Sciences, Indiana University, Bloomington, IN USA

**Keywords:** Contact sport, Cervical spine, Musculoskeletal injury

## Abstract

**Background:**

While morphological changes to the cervical spine have been observed for over 40 years in response to contact sport participation, little is known about the secondary effects of the cervical impairment on future musculoskeletal injury and disability.

**Objectives and design:**

A scoping review was performed to discuss the relationship between contact sport participation and morphological changes to the cervical spine. Moreover, the correlation between morphological changes in the musculoskeletal structures of the cervical spine and resultant deficits in cervical sensorimotor and neuromotor function are discussed. Lastly, how alterations in cervical sensorimotor function may affect overall risk of musculoskeletal injury is discussed.

**Methods:**

The scientific literature was searched in PubMed, Sport Discus, and Web of Science pertaining to contact-sport athletes and/or cervical pathology and the cervicocephalic network. The Asksey and O’Malley’s framework and PRISMA for Scoping Reviews were used to conduct and report the following review. Included articles were grouped into three categories: (1) Morphological changes to the cervical spine in contact sport athletes. (2) The role of the neuromotor pathways of the cervical spine in maintenance of postural tone and coordination of the extremities. (3) The correlation between altered cervical sensorimotor function and a resultant increase in musculoskeletal injury risk.

**Results:**

Our search identified 566 documents, of which 405 underwent full-text screening, resulting in 54 eligible studies for the review. Widespread cervical sensorimotor dysfunction was observed in contact sport athletes. Independently, cervical sensorimotor function was demonstrated to play a critical role in postural control and limb coordination. However, limited research exists exploring the interaction between contact sport participation and altered cervical sensorimotor function, as well as an associated increase in musculoskeletal injury risk.

**Conclusions:**

Limited evidence exists linking cervical injury and/or observed deficits in cervical sensorimotor and neuromotor function to musculoskeletal injury risk. Longitudinal studies combining imaging measures (e.g., MRI, DEXA), cervical functional test, and prospective injury risk are needed to further explore the correlation between resultant cervical sensorimotor deficits following contact sport impacts and future musculoskeletal injury risk.

**Supplementary Information:**

The online version contains supplementary material available at 10.1186/s12998-022-00458-w.

## Background

For over 40 years, morphological changes to the musculoskeletal structures of cervical spine have been observed in contact sport athletes [1–4]. These changes are thought to be a result of mechanical loads to the musculoskeletal structures of the cervical spine, which modulate up to 80% of the mechanical forces applied to the body following sports-related repetitive head impacts (RHI) [5]. RHI are thought to play a catalytic role in early degeneration of the cervical spine, including cervical stenosis, degenerative disk disease, osteoarthritis, and spondylosis/spondylolisthesis [3, 6–9]. Moreover, acute musculoskeletal injuries to the cervical spine, such as cervical strains, fractures, and sprains, frequently develop into more chronic disability lasting up to 24 months and beyond [10, 11]. Understanding the relationship between RHI and degeneration of the musculoskeletal components of the cervical spine is critical to weigh the risks and benefits of contact sport participation [12].

In addition to the morphological changes to the musculoskeletal structures of the cervical spine, alterations in the neuromotor and sensory pathways of the cervical spine have been identified in contact sport athletes. For example, alterations in cervical joint position error, cervical strength, and cervical range of motion have been observed in individuals with a history of contact sport participation as well as following exposure to instrumented RHI [13–16]. Preliminary studies have subsequently linked decreased cervical joint position error, cervical weakness, decreased static posture, and inhibited trunk activation to an increased risk of suffering future injury (e.g., lateral ankle sprain, anterior cruciate ligament tear, concussion) [17–19]. These studies highlight preliminary evidence of a relationship between RHI and future injury through alterations in the neuromotor pathways and decreased functional capacities of the cervical spine. The neuromuscular and sensory pathways of the cervical spine and brain form the cervicocephalic connection, which serves as a vital component of postural tone and neuromuscular coordination [20–23]. Cervical afferent signals work synergistically with efferent motor signals to coordinate the position of limbs throughout sports-related task [20–22, 24].

Despite frequent observations of cervical deficits in contact sport athletes, little research has focused on how those morphological changes impact the complex neurological connections between the brain and the cervical spine, which form the cervicocephalic connection. Moreover, limited research has investigated the connection between cervical deficits and prospective musculoskeletal injury risk, despite the strong documented relationships between the cervicocephalic pathways and neuromotor coordination of the limbs [20, 21, 25].

Due to the paucity of research in understanding the direct causal relationship between RHI and subsequent increased cervical dyskinesia, we established the following purposes for this scoping review: (1) Summarize evidence detailing acute and chronic morphological changes to the cervical spine in response to exposure to contact sport participation. (2) Briefly describe the cervicocephalic network and its role in proper maintenance of head position, postural tone, and coordination of the extremities. (3) Summarize existing research pertaining to a theoretical likelihood of an increase in injury risk due to altered cervical sensorimotor function in a useful way for practitioners and other relevant stakeholders. (4) Identify gaps in existing literature detailing the connection between contact sport participation, cervical disability, decreased cervical sensorimotor function, and consequential resultant changes in musculoskeletal injury risk.

## Methods

We conducted a scoping review, as this approach is superior in addressing an exploratory research question [26–28]. Working within the preferred reporting items for Systematic Reviews and Meta-Analyses extension for Scoping Reviews (PRISMA-ScR), we followed the framework of Arksey and O’malley [26], incorporating adaptations from Levac et al. and Joanna Briggs Institute [28, 29]. At the initiation of this review, there were no databases for registering a *priori* scoping review strategies.

### Stage 1: Identify the research question(s)

Research Questions:

1. *What is known about damage to the cervical spine and neuropathways in response to contact sport participation?*
2. *What is the role of cervical sensorimotor function in posture maintenance and neuromuscular control of the extremities?*
3. *What is the relationship between changes in cervical sensorimotor, neuromuscular control deficits, and increased risk of injury?*

### Stage 2: Identify relevant studies

*Inclusion Criteria*

- Articles of any study design or source (peer-reviewed, grey literature, reviews)
- All age groups and sexes
- Contact sport athletes (e.g., hockey, lacrosse, American football, soccer, rugby, Australian football, wrestling)
- Musculoskeletal injury, head impacts, and or degenerative condition affecting the cervical spine

*Exclusion Criteria*

- Abstracts where no full-text was available
- Case reports
- Not published in the English language
- *Step 1* Initial limited search

An initial limited search (May 2021) was conducted. The search terms used were “contact sport” AND “cervical spine”. A total of 122 articles were reviewed. Twenty-two studies were included, with the references from those studies being reviewed for further relevant papers. The entire search strategy is detailed in Additional file 1: Appendix A.

- *Step 2* Identify key words and index terms

The title, abstract, and index terms used to describe the articles identified in step 1 were analyzed. The first search focused on prevalence of cervical pathology in contact sport athletes. Separately, articles that demonstrated the connections between cervical kinesthesia and postural control unrelated to contact sport participation were searched. Lastly, a search for studies that combined these two concepts was performed.

For the preliminary search ‘Cervical Spine’ and ‘Sport’ were set as the primary research terms. Secondary search terms (Additional file 1: Appendix A) were then added along with Boolean terms AND and OR. This process was then repeated with search terms ‘cervical kinesthesia’ and ‘posture’ to stratify evidence related to cervicocephalic connection. Lastly, ProQuest database as well as google scholar were searched to explore potential grey literature.

- *Step 3* Further Searching of references and citations

A final search of the reference list of identified articles was performed. A completed final search strategy is shown in Additional file 1: Appendix A.

### Stage 3: Study selection

**Fig. 1:**
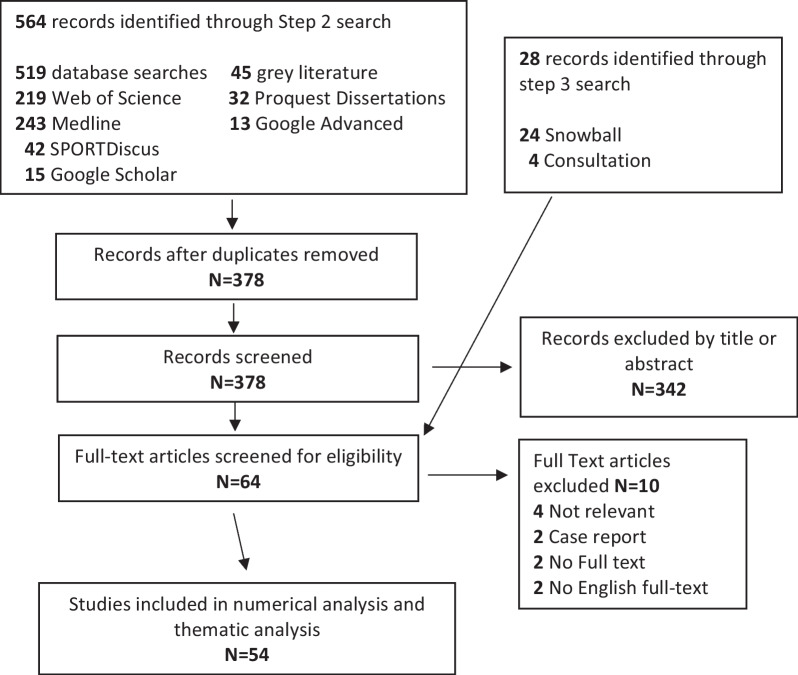
Scoping review flow chart

Titles and abstracts were evaluated by two reviewers (KC and KK). A third reviewer (JK) completed a random sample of 20% of the titles and abstracts as a quality check and achieved concordance > 97% regarding the decision of each article [28–30]. Additional file 1: Appendix B details the selection and screening criteria. All titles and abstracts were reviewed independently, and any disagreement was discussed and the full-text was reviewed. A Prisma flow diagram (Fig. 1) details articles excluded at each step.

### Stage 4: Charting the data

KC and KK independently trialed the data extraction form which included: author(s), year of publication, aims/purpose, participants, methodology, outcomes, intervention type, and key findings on 10 selected studies. Following this step, KC extracted data from 90% of included studies, and KK extracted data from 10% of the studies. Following data extraction, KC reviewed those studies extracted by KK while KK reviewed 10% of the studies that KC extracted. Ninety-five percent overall concordance was established, in agreement with previously published scoping reviews thus, no further cross-checking was performed [28, 29]. Table 1 presents a summary of each article identified in the scoping review.

**Table 1:** Details of identified studies

Author	Participants	Study design	Year	Intervention	Outcomes	Key findings
*Theme 1. Cervical spine and contact sport participation* *1.1 Epidemiology of cervical injuries*						
Mueller [33]	N/A	Literature review	1998	N/A	N/A	Dramatic reduction in fatalities 1975 through 1994 1976 rule change that prohibited initial contact with the head and face when blocking and tackling
Cantu [31]	183 High School 29 Collegiate 7 Professional	Retrospective review of injury records	2003	N/A	Catastrophic spine injuries in football	Incidence rate of 0.52 in high school, 1.55 in college, and 14 in professional football for every 100,000 participants A 270% reduction in catastrophic injuries was observed following 1976 rule change
Fuller [39]	Professional Soccer athletes	Case control	2005	N/A	Cervical Injury rates	Most frequent injuries were contusions (53%), lacerations (20%), and concussions (11%) The incidence of all head and neck injuries was 12.5/1000 player hours (men 12.8, women 11.5) and 3.7 for lost-time injuries (men 3.5, women 4.1) Frequent mechanisms of injury involved aerial challenges (55%) and use of the upper extremity (33%) or head (30%)
Nilsson [40]	26 European soccer teams between 2001/2002 -2009/2010	Prospective cohort study	2013	Professional Soccer participation	Injury rate (number of time loss injuries per 1000 h)	136 head and neck injuries were recorded (2.2% of all injuries) Injury rate was 0.17 (0.06 concussions) per 1000 h 20-fold higher rate of head and neck injury during match play compared with training (rate ratio[RR], 20.2; 95% [CI] 13.3–30.6) and a 78-fold higher rate of concussions (RR, 78.5; 95% CI 24.4–252.5)
Hutton [32]	N/A	Systematic review	2016	N/A	Incidence catastrophic cervical injuries	Among Rugby Union players, incidence of catastrophic cervical spine injury (CCSI) was 4.1 per 100,000 player-hours Among NFL players, the CCSI rate was 0.6 while collegiate rate ranged from 1.1 to 4.7 per 100,000 player-years CCSI rate of 1.4–7.2 per 100,000 player-years
Meron [36]	National High School Sports-related Injury Surveillance system	Retrospective review of records	2017	N/A	Cervical spine injury rates	1080 cervical spine injuries were reported during 35,581,036 athletic exposure (AE), resulting in an injury rate of 3.04 per 100,000 AE Cervical spine injuries were highest in football (10.10), wrestling (7.42) and gymnastics (4.95) Muscle injuries were the most common (63.1%), followed by nerve injuries (20.5%) The most common mechanisms of injury were contact with another player (70.7%) and contact with playing surface (16.1%)
Simmons [37]	Ice hockey NCAA Injury Surveillance Program 2009–2014	Descriptive epidemiology study	2017	N/A	Head and neck Injury rates per 1000 AE	Injury rate was higher in men than in women (1.75 vs. 1.16/1000 AE; CI = 1.25, 1.84) The most common head and neck injury was concussion; most concussions occurred in men's competitions from player contact while checking (25.9%)
Williams [38]	3 NCAA Division I universities from 2007 to 2012	Descriptive study	2017		Head and neck injury rates per 10,000 AE	Overall injury rate was 35.2 per 10,000 athletic exposure (AE)s Rates for initial and subsequent injuries were 31.7 and 45.3 per 10,000 AEs, respectively, with a rate ratio (RR) of 1.4 for rate of subsequent injury vs rate of initial injury (95% CI 1.1–1.9) Subsequent injuries to the head, neck, and face were nearly double the rate of initial injury to same site (10.9 per 10,000 AEs, RR = 2.0; 95% CI 1.1–3.5)
Chung [34]	Collegiate football players in the NCAA database	Descriptive study	2019	N/A	Cervical spine injury rates	300 cervical Injuries were identified in the data representing an estimated 7496 total cervical spine injuries extrapolated from the observed population to entire NCAA Injury rate of 2.91 per 10,000 AEs Most common was stinger with 1.8/10000 AEs and cervical strains with 0.8/10000 AEs Injuries were highest among defensive players
Lee [35]	High school and collegiate athletes from national sport injury surveillance databases	Retrospective record review	2019	N/A	Cervical injury counts and rates/10,000 AE	The NCAA database reported 49 cervical muscle strains (rate = 0.96/10000 AEs), (57.1%) were time loss injuries (rate = 0.55/10, 000 AEs) High School databases reported 184 cervical muscle strains (rate = 1.66/10, 000 AEs), of which 33 (17.9%) were time-loss injuries (rate = 0.30/10,000 AEs) -The overall injury rate was lower among collegiate athletes than high school (injury rate ratio = 0.58; 95% CI = 0.42, 0.79)
*Theme 1. Cervical spine and contact sport participation* *1.2 Morphological changes to the cervical spine in contact sport athletes*						
Sortland [3]	43 national soccer players from Norway 43 matched control	Cross-sectional	1982	Exposure to professional soccer	Spinal axis, healed fractures, cervical degenerative changes	Compared with men of the same age group the onset of degeneration was 10–20 years earlier and the frequency of degeneration was significantly higher in retired professional soccer players Degenerative changes were not especially high in among individuals who reported higher heading frequency but this group had a higher frequency of subjective complaints and clinical findings such as reduced cervical movements
Kuman [43]	30 Athletes	Cross-sectional observational	1986	Treatment of rest and traction versus referral for surgery	Time to recovery radicular symptoms vs no radicular symptoms	60% of radicular signs and symptoms were from the 4th and 5th cervical intervertebral space Roentgenographic changes were most common at the 4th and 5th cervical root Most cases responded favorably to conservative treatment of traction and rest Patients who presented with radicular signs and symptoms required up to 5 months to return while athletes with no radicular signs returned in less than 3 weeks
Tysvaer [47]	69 active soccer players and 37 retired Norwegian national team members	Cross sectional	1992	Exposure to professional soccer	Computerized tomography electroencephalogram (EEG)	Head injuries account for between 4 and 22% of soccer injuries There were fewer abnormal EEG changes among typical 'headers'(10%) than among 'nonheaders' (27%) One-third of the players were found to have central cerebral atrophy and 81% to have from mild to severe neuropsychological impairment The radiological examination of the cervical spine revealed a significantly higher incidence and degree of degenerative changes than in a matched control group
Torg [46]	5 groups of individuals based on graded amount of years of exposure to football	Descriptive study	1996	Exposure to football	Torg ratio	A torg ratio (Diameter of spinal canal/diameter of the vertebral body) of 0.80 or less had a high sensitivity (93%) for transient neurapraxia Developmental narrowing of the cervical canal in a stable spine does not appear to predispose an individual to permanent catastrophic neurological injury and therefore should not preclude an athlete from participation in contact sports
Quarrie [45]	N/A	Review article	2002	N/A	N/A	Majority of injuries occur early in the season, when players are lacking both practice and physical conditioning for the physical contact phases of the sport Hookers and props have been at disproportionate risk of cervical spine injury, predominantly because the scrum was the phase of play most commonly associated with spinal injuries
Kartal [8]	15 veteran, 15 current, 28 age matched controls soccer players	Cross-sectional descriptive study	2004	Exposure to soccer	Cervical strength Cervical RoM Cervical X-ray Spinal cord compression	Degenerative changes were prominent in veteran players, and the sagittal diameter spinal canal at C5 to C7 was lower when compared to active players and controls Magnetic resonance findings of degeneration were more prominent in soccer players when compared to their age-matched controls A tendency towards early degenerative changes exists in soccer players
Bailes [45]	Ten contact sport athletes	Cross-sectional Observation	2005	N/A	X-ray and CT with dynamic studies	The occurrence of TSCI is not uncommon in athletes involved in contact sports Transient spinal cord injuries appear among those in yet whom radiographic studies are normal, and those with cervical stenosis, the later is the most difficult management group
Mehnert [9]	N/A	Review	2005	N/A	N/A	Existing studies of long-term effects suggest a predisposition to degenerative changes of the cervical spine Further research in this area is needed with studies that assess biomechanical forces under simulated play conditions and control for impacts and stresses to the neck and spine that occur from non-heading activity
Ivancic [48]	10 cadavers	Pre-post Experimental	2013	Crash simulation	Intact and postimpact flexibility test, axial torque and lateral bending	Multidirectional instability of the upper cervical spine caused by atlas and dens fractures was evidenced by increases up to 53% in cervical flexion and extension due to impacts Increases in extension range of motion were 14.9 degrees in the upper cervical spine and 24.9 degrees (*p* < 0.05) at the middle cervical spine and in flexion at C7/T1 were 25.6 degrees
Brauge [42]	101 former rugby players (mean age 40.4) 85 aged matched controls	Cross-sectional	2015	Participation as a professional rugby athlete	Japanese orthopaedic questionnaire Neck disability MRI	Rugby players complained of chronic neck and decreased mobility pain more frequently (51 of 101 vs. 27 of 85, *p* = 0.01) Rugby players had a narrower vertebral canal(0.88 ± 0.167 cm vs. 0.99 ± 0.130 cm, *p* = 0.007) and more foraminal stenosis (*p* = 0.01) Rugby players had more often undergone surgery for a degenerative condition than controls (10 cases vs. 0, *p* = 0.0021)
Trewartha [49]	N/A	Literature Review	2015	N/A	N/A	Scrummaging leads to premature chronic degeneration of the cervical spine Biomechanical studies of rugby scrummaging confirm that scrum engagement forces are high and multiplanar, but can be altered through modifications to the scrum engagement process to control engagement velocity The incidence of acute injury associated with scrummaging is moderate but the risk per event is high
Ndubuisi	204 symptom free adults, 21–50 years of age	Cross-sectional	2017	Exposure to active leisure contact sports	Space available for the Cord (SAC)	SAC at C3-4 was 4.39 ± 0.28 mm contact sport group and 4.90 ± 0.30 mm in controls (*p* = 0.036) and at C4-5 was 4.16 ± 0.27 mm contact sport group and 4.56 ± 0.35 mm (group B) Significant effect of contact sports (*p* = 0.005), sex (*p* = 0.001), and age (*p* = 0.0001) were observed in relation to SAC
*Theme 1. Cervical spine and contact sport participation* *1.3 Return to play considerations*						
Cantu [51]	N/A	Informational	1998	N/A	Clinical criteria for diagnosis of cervical stenosis	Spinal stenosis can't be defined by bone measurement alone as this fails to control for dural compression Patients with functional spinal stenosis recovery far less frequently than those who have structural narrowing of the spine as measured by radiography Radiography is critical for initial work up to clear subluxation or fracture when symptoms are present
Okonkwo [58]	N/a	Review	2003	N/A	N/A	Two million persons suffer a head injury each year in the United States; of these, approximately 350,000 are sports- and recreation-related head injuries Between 12,000 and 15,000 cases of spinal cord injury occur each year in the United States, of which 10–15% are sports-related Traumatic brain injury is the most common cause of death in persons under 45 in the western world
Torg [59]	N/A	Systematic review	2009	N/A	N/A	The overriding principle regarding the return to football or any collision activity should be that the individual is asymptomatic, pain-free, and neurologically intact and have full cervical strength and range of cervical motion Any injury to C1-C2 is an immediate contraindication to contact sport participation
Chao [52]	N/A	Review article	2010	N/A	N/A	Catastrophic cervical injuries at are rare and account for less than 3% of cervical spine injuries Transient neurological episodes are estimated at 7/10,000 and typically resolve in 10–15 min but may last up to days Most frequent mechanism of brachial plexus injury is traction and are associated with athletes with a higher rate of cervical stenosis, disk disease and other degenerative conditions
Dailey [53]	N/A	Clinically based systematic review	2010	N/A	N/A	Weak recommendation that patients with transient neuropraxia and radiographic evidence of cervical canal compromise should be withheld from participation in contact sports Strong recommendation that patients with transient neuropraxia without radiographic evidence of cervical stenosis can return to full sports activities
Kepler [55]	N/A	Expert opinion	2012	N/A	N/A	Benign injury types such as isolated spinous process fractures or compression fractures can be treated with immobilization and typically do not preclude return to play once healed Complex injuries must be evaluated based on spinal stability, need for fusion, and the number of levels fused if necessary; fusion of 3 or more cervical levels is a contraindication to return to play Players with a third stinger in a single season or a recurrent transient quadriparesis must undergo imaging to rule out stenosis and parenchymal injury; return to play is dependent on resolution of symptoms and severity of episode
Mcana [57]	N/A	Commentary	2014	N/A	N/A	Cervical spondylolysis, unlike lumbar spondylolysis, is an exceedingly rare only 150 have been reported Limited knowledge is known about RTP guidelines following cervical spondylolysis
Joaquim [54]	N/A	Systematic Review	2016	N/A	Relief of symptoms, RTP, career length after surgery, and permanent neurological deficits	Return to play is safe for athletes who are asymptomatic after disk fusion for cervical radiculopathy due to disc herniation Surgical treatment may provide a higher rate of return to play for these athletes than nonsurgical treatment Cervical cord signal changes may not be an absolute contraindication for return to play in neurologically intact patients Cervical contusions secondary to cervical stenosis may be associated with a worse outcome and a higher recurrence rate than those secondary to disc herniation
*Theme 2: Cervical afferents and postural control* *2.1 Artificially induced afferent cervical dysfunction alters neuromotor control and posture maintenance*						
McLain [23]	21 cervical facet capsules, taken from three normal human subjects	Cross-sectional	1994	N/A	Mechanorece-ptor count Nociceptive nerve ending count	The presence of mechanoreceptive and nociceptive nerve endings in cervical facet capsules proves that these tissues are monitored by the central nervous system Protective muscular reflexes modulated by these types of mechanoreceptors are important in preventing joint instability and degeneration
Allum [20]	4–10 controls 3–7 bilateral labyrinthine deficient patients (depending on experiment)	Cross-sectional	1997	Labyrinthine deficient vs normal control	EMG Head acceleration	Head velocities observed during balance corrections depend to a large extent on the movements of the head-neck mass-viscoelastic system whose properties could be altered by co-contracting the neck muscles For experiments involving stance perturbations, much of the corrective response in neck muscles appeared to be triggered by trunk and leg proprioceptive signals Cervical reflexes modulate the amplitude of functionally stabilizing responses and dampen mechanically induced instability of the head and neck
MalmstrÃm [64]	16 healthy subjects	Cross-sectional experimental	2017	Disturbance of cervical proprioception by vibration	Spatial body position	Significant differences were seen in posturography between no vibration (628 mm or 25.1 mm/s) relative to each vibration condition When vibration was applied on the left-sided muscles, rotation to the right was induced (*p* = 0.005) Cervical proprioception is a critical component of internal spatial orientation and postural control
*Theme 2: Cervical afferents and postural control* *2.2 Cervical pathology is linked to altered neuromotor control and poor posture maintenance*						
Karlberg [63]	17 with cervicogenic dizziness 17 healthy controls	Randomized controlled trial	1996	Physical therapy to reduce symptoms	Vibration-induced body sway, intensity and frequency of neck pain	Neck pain patients manifested significantly poorer postural performance than did healthy subjects (0.05 > *p* > 0.0001) Physiotherapy significantly reduced neck pain and intensity and the frequency of dizziness (*p* < 0.01), and significantly improved postural performance (0.05 > *p* > 0.0007)
Sjostrom [66]	25 whiplash participant 170 healthy age-matched control participants	Cross-sectional	2003	Whiplash vs healthy	Trunk sway	Greater trunk sway for stance tasks and for complex gait tasks that required task-specific gaze control such as walking up and down stairs or walking while turning the head
Treleaven [68]	100 whiplash participants (50 dizziness/50 no dizziness) 50 healthy controls	Prospective observational design	2005	Whiplash with and without dizziness vs healthy control	Clinical test for sensory Interaction in balance	Energy of the sway signal for comfortable stance tests was significantly greater in the group with dizziness compared with the group without dizziness Subjects with dizziness were significantly less able to complete the test than subjects without dizziness and controls after controlling for medications, compensation, anxiety or age and are likely to be due to disturbances to the postural control system possibly originating from abnormal cervical afferent input
*Theme 3: Damage to cervical afferents and increased risk of injury*						
Treleaven [67]	N/A	Masterclass	2008	N/A	N/A	The importance of the cervical reflex connections on postural control can be understood by observing changes in postural sway in response to artificial disturbances to the cervical afferents in asymptomatic individuals Dysfunction of the cervical receptors following injury to the cervical spine can alter afferent input subsequently changing the integration, timing and tuning of sensorimotor control of the extremities Afferent information from the cervical receptors can be altered via a number of mechanisms such as trauma and or functional impairment of the receptors, changes in muscle spindle sensitivity and the vast effects of pain at many levels of the nervous system
Freppel [62]	17 patients with degenerative cervical spine disease 31 healthy controls	Pre-test, post-test	2013	Surgery to correct either herniated disk or cervical degenerative disease	Displacement in center of foot pressure Static posture tests	C ontribution of visual input to postural control is reduced in a dynamic visual environment where cervical spine diseases is present The relative importance of visual and proprioceptive inputs to postural control varies according to the type of pathology and surgery tends to reduce visual contribution mostly in the spondylosis group
Treleaven	140 whiplash patient	Randomized controlled trial	2016	whiplash vs healthy control	Static and dynamic clinical balance tests and cervical joint position error	Between and within group comparisons suggest that physiotherapist led neck exercise groups had advantages in improving measures of dizziness compared with the general physical activity group, although many still complained of dizziness and balance impairment
Wannaprom [69]	30 adults withchronic neck pain 30 healthy control	Cross-sectional	2018	neck pain vs healthy control Vibration of suboccipital muscles	Balance in a comfortable stance and timed gait test using a 10 m walk test	At baseline, neck pain participants had greater postural sway, and slower gait speed than healthy controls (*p* < 0.001) Immediately after vibration, neck pain participants displayed decreased postural sway, and increased gait speed (*p* < 0.001) Neck muscle vibration improved standing balance and gait speed in participants with neck pain but reduced performance in healthy controls
Reddy [65]	132 subjects with cervical spondylosis (CS) and 132 healthy age-matched control	Cross sectional	2019		Cervical JPE testing, neck pain assessment,	Cervical spine injury subjects (CS) showed significantly larger cervical joint position sense error than healthy control subjects in all the directions tested(flexion, extension, right and left rotation) with a *p* value (< 0.001) Comparing all the movement directions in the CS and healthy control groups, the cervical joint position sense were largest in cervical extension (CS groups = 8.28° ± 1.80°; healthy group = 4.48° ± 1.26°) with standard error of measurements of 0.21° and minimal detectable change of 0.48°
Carrick [61]	575 concussion patients 60 healthy controls	Repeated measures experimental	2020	Concussed vs healthy Changes in head position during static posture assessment	Computerized dynamic posturographic measurement	Postural stability scores are correlated with changes in head position in subjects following a concussion The position of the head and neck induced by statically maintained head turns is associated with significantly lower stability scores than the standardized head neutral position in Post-concussion Syndrome subjects but not in normal healthy controls Head positions on the neutral plane provide novel biomarkers that identify and differentiate subjects suffering from persistent post-concussion symptoms from healthy normal subjects
Hammerle	48 post-concussion patients with dizziness	Retrospective record review	2019	Vestibular vs. cervical therapy	Improvement in dizziness	Patients who received cervical specific therapy were 30 times more likely to report improvement in dizziness symptoms compared with those who received vestibular therapy alone (adjusted odds ratio: 30.12; 95% confidence interval 4.44–204.26, *p* < 0.001) when cervical spine symptoms were present
Hides [72]	190 Male rugby players 47 reported history of concussion	Prospective cohort study	2017	History of reported concussion	Balance Vestibular function Cervical Joint position error Trunk muscle function	A history of concussion was related to changes in size and contraction of trunk muscles 22 (11.6%) players sustained a head/neck injury during the playing season of which 14 (63.6%) players had a previous history of SRC Five risk factors in total were identified (cervical joint position error, history of concussion, and 3 measures of trunk muscle function) athletes with 3 or more were 14 times more likely to suffer a neck/head injury (sensitivity 75% and specificity 82) then players with 2 or fewer risk factors
Hides [19]	54 Professional rugby league players 14 suffered concussion	Prospective cohort study	2017	Pre/Post Concussion	Trunk muscle imaging Cervical joint position error Balance assessment	Significant decreases in sway velocity and increased size/contraction of trunk muscles, were identified following concussion Whilst not significant overall, large inter-individual variation of test results for cervical proprioception and the vestibular system was observed Preliminary findings Post-concussion suggest an altered balance strategy and trunk muscle control with splinting/over-holding requiring consideration as part of the development of appropriate physiotherapy management strategies
Howell [27]	N/A	Scoping review	2018	N/A	N/A	Based on existing literature, athletes appear more likely to sustain a musculoskeletal injury in the year after sustaining a concussion There are no known mechanisms for the increase in lower extremity injury following concussion, but one contributing factor may be that continued neuromuscular control deficits exist for a longer period than standard clinical tests are equipped to identify
Abdelkader [71]	45 (18–40 years old)	Repeated measures	2020	Fatigue	Neck Reposition Error Postural control (biodex balance system multidirectional reach test)	Declines in postural stability were correlated with increased cervical joint position error Subjects suffering from cervical muscles fatigue are vulnerable to neck proprioception deficits and postural instability Clinicians and patients should avoid overload fatigue of the cervical muscles because it affects overall postural balance, neck proprioception & righting reaction
Cheever [13]	40 female colligate club soccer athletes	Repeated measures pre-test post-test design with 4 groups	2020	Instrumented head impacts Fatigue workout	Cervical joint position error test, Neck Disability Index	A 65%,54%,and49% increases in cervical joint position error were observed following soccer heading, fatigue only, and soccer heading + fatigue interventions, respectively. Meanwhile, the controls who did not head soccer balls or complete the fatigue protocol saw a 6% decrease in neck position error No difference between fatigue group and head impact group
Cheever [14]	27 contact sport athletes 20 controls	Cross-sectional	2021	History of contact sport participation	Cervical strength Cervical joint reposition error Neck Disability Index	Amateur sport athletes with a history of contact sport exposure exhibited 25.2% more total neck reposition error and 24.6% more maximum neck reposition error than athletes with no history of contact sport participation S/S number (r2 = 0.12, F(2,44 = 6.2, *p* = 0.017) and S/S severity (r2 = 0.14, F(2,44) = 5.6, *p* = 0.02) were significantly correlated with total neck reposition error
Peng	N/A	Narrative Review	2021	N/A	N/A	Main problem in patients with neck pain is the impairment of cervical proprioception, which subsequently leads to cervical sensorimotor control disturbances Experimental neck muscle pain induced by injection of hypertonic saline results in significant inhibition of the activation of painful muscles suggesting chronic neck pain may cause structural and functional impairment of cervical muscles leading to excessive activation of mechanoreceptors in degenerative cervical discs and facet joints producing a large number of erroneous sensory signals

*NCAA* National Collegiate Athletic Association

### Stage 5: Collating, summarizing, and reporting the results

The methods employed throughout this protocol enabled us to collate existing knowledge and summarize the data in the following ways

1. A numerical and descriptive analysis, mapping of data within a scoping review flow chart.
2. An inductive thematic summary and analysis describing to the research questions and aims, based on the analysis on Braum and Clarke’s six-phase framework.

## Results

### Descriptive analysis

Our initial search identified 564 documents. Of these, 519 were within databases/search platforms and 45 from grey literature (Fig. 1). After duplicates were excluded, 378 records remained. An additional 28 potential studies were identified during step 3. In total, 406 articles underwent screening, and 315 records were excluded following abstract screening. An additional 37 were removed after reading full text. As a result, the scoping review identified 54 eligible studies (Fig. 1).

### Included studies by year of publication

**Fig. 2:**
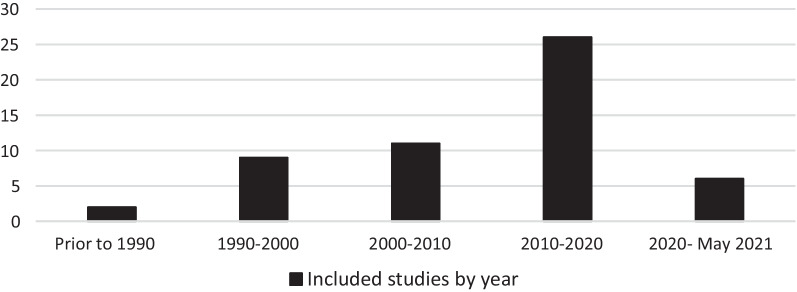
Included studies by year of publication

As consistent across the wider bibliometric trends, a substantial chronological increase in the number of relevant papers was identified over the previous 50 years (Fig. 2).

### Type of study

#### Study design

**Fig. 3:**
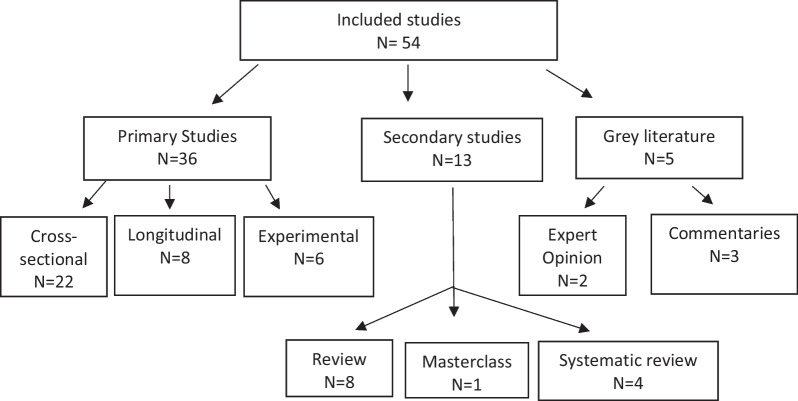
Taxonomy of research designs for included literature

The studies varied in terms of study design and primary focus. No formal quality assessment of included studies was performed, as scoping reviews are intended to provide a map of what evidence has been produced as opposed to seeking only the best available evidence to create policy or answer evidence-based practice questions [26, 30]. A taxonomy of research designs included in this scoping review is shown in Fig. 3.

### Theme of the study

Each included study fit into one of 3 themes which were further divided up into subthemes to accomplish the 4 purposes outlined in the introduction.

#### Theme 1: Cervical spine and contact sport participation

Thirty-three articles detailed the relationship between contact sport participation and the cervical spine. These articles present the evidence relating to the effects of contact sport participation on the cervical spine congruent with our first purpose. These studies provided consistent evidence of damage to the structures of the cervical spine responsible for maintaining the cervicocepahlic connection. Three secondary themes, including: (1) epidemiology of cervical injuries; (2) morphological changes to the cervical spine; and (3) return to play considerations emerged.

##### Epidemiology of cervical injuries in contact sport athletes

Three studies identified 223 catastrophic cervical injuries [31–33]. Representing an incidence rate of 0.52 in high school, 1.55 in college, and 14.00 in professional football for every 100,000 participants [31, 33]. Five epidemiological studies explored the prevalence of acute head and neck injuries in contact sport athletes [34–38]. Among the collegiate population, an estimated 7496 total cervical spine injuries between 2009–2010 to 2013–2014 were extrapolated from a smaller observed population, creating an estimated injury rate of 2.91 per 10,000 athlete exposure (AE) [34]. Stinger was the most common cervical injury (1.8/10,000 AE), followed by cervical strains (0.8/10,000 AEs) [34]. Among high school students, time loss cervical spine injuries were estimated at 3.04 per 100,000 AEs [36]. The most frequent mechanism of a cervical injury was contact with another player (70–85%) followed by contact with the ground (15–20%) [35, 36].

Two prospective studies explored head and neck injuries in professional soccer athletes [39, 40]. The incidence rate was 0.17–12.5/1000 player hours (men 12.8, women 11.5) and 3.7/1000 player hours for lost-time injuries (men 3.5, women 4.1) [39]. Mechanisms of injury involved aerial challenges (55%) and the use of the upper extremity (33%) or head (30%). [39]

##### Morphological changes to the cervical spine

Ten studies identified degenerative changes such as cervical spinal stenosis, degenerative disk disease, temporary paralysis, spondylolisthesis, and neck pain 10–20 years prior to aged-matched controls among contact sport athletes [3, 8, 41–47]. Lastly, three studies reviewed the experimental biomechanical stresses placed on the cervical spine in response to mechanical head loads during contact sport participation [9, 48, 49]. In these studies, multidirectional instability in the upper cervical spine was observed following simulated head impacts.

##### Return to play considerations

Ten studies explained principles guiding returning to play which included: asymptomatic, pain-free, neurologically intact, full strength and full cervical range of motion [50–59]. The primary concerns included reticulating pain, damage to C1 or C2, and instability [59, 60]. However, observations of increased risk of musculoskeletal injury following a concussion, portend the importance of caring for surrounding structures [19, 27].

The two most frequent concerns for disqualification were spinal stenosis and brachial plexus injury. Brachial plexus injury has an estimated 87% reoccurrence rate and caution should be taken to return those athletes with reoccurring radicular pain [53].

#### Theme 2: Cervical afferents and postural control

A total of twelve studies demonstrated the role cervical afferents play in upright posture and control of locomotion [11, 20, 23, 61–69]. These articles describe the cervicocephalic network and the role it plays in proper maintenance of head position, postural tone and coordination of the extremities congruent with the 2nd purpose of this review. Cervical afferents such as mechanoreceptors and nociceptive nerve endings are prevalent in cervical facet capsular tissue [23]. High densities up to 200 muscle spindles per gram compared to the first lumbrical in the thumb which has a mere 16 muscle spindles per gram [67].

##### Artificially induced afferent cervical dysfunction alters neuromotor control and posture maintenance

Three studies demonstrated how vibration and/or using anesthetic injections to simulate cervical dysfunction could cause severe ataxia and disequilibrium [64, 67, 69]. Malmstrom et al. [64] demonstrated significant increases in error during walking task. Moreover, Wannaprom et al. [69] reported increased postural sway and slower gait speeds under a vibration condition. Allum et al. [20] compared healthy controls to individuals with labyrinthine deficiency to isolate the contributions of the vestibular and cervical afferents in head control revealing head velocities during balance corrections were highly dependent on the movements of the head-neck mass-viscoelastic system. The cervico-collic reflex was also found to modulate the amplitude of functionally stabilizing responses and dampen mechanically induced instability of the head and neck.

##### Cervical pathology is linked to altered neuromotor control and poor posture maintenance

In addition to experiments in healthy controls, alterations in postural maintenance and locomotion, as well as cervical afferent processing following cervical pathology have been observed [11, 61–68, 70]. Patients with neck pain and dizziness demonstrate decreased postural stability [11, 63], as well as decreases in cervical sensorimotor function [62]. Specifically, cervical joint position error was significantly correlated with greater levels of neck pain and extent of cervical degeneration [62]. These findings have been replicated several times [11, 62, 65]. Separately, changes in cervical joint position error have also been correlated with altered postural stability [71].

#### Theme 3: Damage to cervical afferents and increased risk of injury

In considering the 3rd aim of the present review 4 studies demonstrate preliminary evidence of a connection between head impacts and altered cervical sensorimotor function [13, 14, 16, 72]. Cheever et al. [14] reported amateur sport athletes with a history of contact sport exposure exhibit 25% more total neck reposition error than controls. A follow up study utilized an acute bout of instrumented head impacts and observed a 65% acute increase in cervical joint position error following soccer heading compared to a 6% decrease among controls who did not complete the heading [13]. A decrease in trunk and head positioning following head impacts has also been observed, suggesting altered balance strategies and trunk muscle activation as regulated by the cervicocephalic network may also manifest following RHI. [72]

Further exploring the 3rd aim two preliminary prospective studies explored the correlation between decreased cervical sensorimotor function and a corresponding increase in musculoskeletal injury risk [13, 19]. In a prospective cohort study performed by Hides et al. [19], five risk factors were identified (cervical joint position error, history of concussion, and 3 measures of trunk muscle function). Athletes with 3 or more risk factors were 14 times more likely to suffer a neck/head injury (sensitivity 75% and specificity 82%) than players with 2 or fewer risk factors. Significantly decreased sway velocity and increased size/contraction of trunk muscles were also correlated with head position sense [19]. A similar study by Cheever et al. [13] found that a combination of sign and symptom severity scores and neck reposition error had robust predictive capability of individual’s future musculoskeletal injury status (AUC = 0.80, *p* = 0.003).

## Discussion

The objective of this scoping review was to present the available evidence of morphological changes to the cervical spine following contact sport participation and to describe a theorized increased risk of musculoskeletal injury due to alterations in cervical sensorimotor function resulting from those morphological changes. Morphological changes to the cervical spine were observed throughout the literature, and associated decreased cervical sensorimotor function were observed in a few instances [13, 14]. Furthermore, preliminary evidence of a correlation between cervical sensorimotor function and secondary musculoskeletal injury was observed. [13, 19]

### Decreased cervical sensorimotor function following morphological changes to the cervical spine and exposure to RHI

A preponderance of studies have demonstrated a relationship contact sport athletes who have a history of scrumming, tackling, and/or head contact with a ball and early development of chronic degeneration of the cervical spine [3, 6, 9, 73]. Separately, acute bouts of soccer heading and a history of contact sport history have been correlated with decreased cervical sensorimotor function [13, 14, 16]. However, while observations of accelerated degeneration and acute damage to the cervical spine have been corroborated in different sports (e.g., soccer, rugby, American football), all studies employed a cross-sectional design, failing to demonstrate a longitudinal correlation [7, 44, 46].

Preliminary findings from a series of instrumented heading studies serve as a potential foundation of a direct link between exposure to RHI and decreased cervical sensorimotor function and posture [13, 74]. Cheever et al. demonstrated a 65% increase in cervical joint reposition error in response to controlled soccer headers [13]. Similarly, decreased balance following instrumented soccer headers has been observed [74]. Both cervical sensorimotor function and balance deficits have been identified independently in patients suffering from chronic cervical pathology and athletes following an acute bout of soccer headers [75–77].

### Cervical sensorimotor dysfunction and increased musculoskeletal injury risk

Previous research studies have demonstrated correlations between decreases in cervical joint position and postural control deficits Independent of contact sport exposure [70–79]. Both patients suffering from persistent cervical symptoms and persistent post-concussive symptoms have demonstrated altered postural control, as well as decreased cervical sensorimotor function [61, 66, 68, 70].

**Fig. 4:**
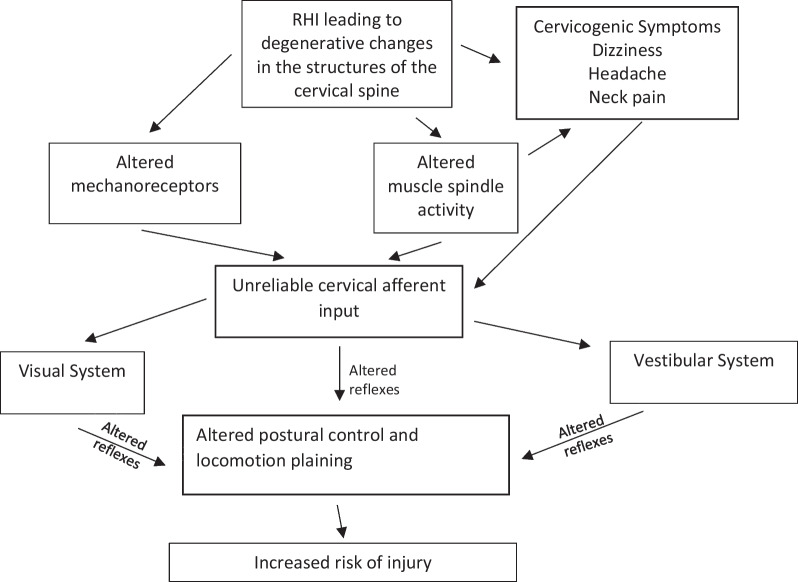
Mapping of potential implications for altered cervical afferent input

A large body of existing literature demonstrates an observed increase in musculoskeletal injury risk in the year after sustaining a concussion [80]. The mechanism for this relationship remains highly contested [27]. However, one leading hypothesis points to observations of lingering neuromuscular control deficits [79]. While not fully explored, altered cervical afferent processing may play a role in this heightened risk. Sensorimotor control of both stable upright position and locomotion relies heavily on cervical afferent information that play a critical role in three reflexes that influence head, eye and postural stability: the cervico-collic reflex, the cervico-ocular reflex, and the tonic neck reflex [67]. These reflexes work alongside the vestibular and visual systems throughout the central nervous system to maintain proper postural control [80]. These connections have been further mapped in Fig. 4 to visually demonstrate the potential connection of morphological and neurological changes to the cervical spine and increased risk of secondary injury.

While findings of impaired sensorimotor function and balance in contact sport athletes are an important step, these movements were all performed at slow speeds, which don’t mirror the pace of sports-related task. Previous experiments exploring the integration of deficient cervical afferent information into the cerviocephalic network were performed in either a static position or walking at a relatively slow steady state (< 2–3 mph) [64, 69]. Athletes frequently travel in excess of 15–20 mph during sprints with individual spikes in limb movement exceeding 8000 degrees/s [81]. No research has been performed in a high velocity movement state to test how variations in postural control and cervical sensorimotor function may be magnified by the high velocity demands of contact sport movement patterns (e.g., dodging a potential tackler, sprinting after an advancing player, or adjusting to a rapid change of direction).

### Knowledge gaps and future research

In line with the 4th aim of the present scoping review several gaps in the available literature were identified (Box 1). Future research should explore the dose dependent effect of RHI on structures of the cervical spine and the causative affect those changes have on musculoskeletal injury risk. Moreover, it would be important to explore potential interventional therapies to reduce the increased risk of injury.

**Box 1:** Relevant research priorities identified

The dose–response effect of RHI and degeneration of cervical spine across different competition levels (youth, high school, college, amateur, professional) and time periods (single exposure, season, career)
Larger prospective cohort studies that utilize measures of cervical sensorimotor function to predict increased injury risk
Large retrospective epidemiological studies utilizing injury sport databases to see if individuals who suffered a neck injury were subsequently more likely to suffer a subsequent secondary injury
The role altered cervical sensorimotor function plays in postural control and coordination of the limbs at speeds more similar to those experienced during sport participation

### Limitations

While scoping reviews are comprehensive, they fall short of exhaustively identifying all relevant literature by balancing the breadth and depth of the desired analysis. Our search was subject to a limited number of contact sports rather than all physical activity involving RHI. Contact sport athletes are different from controls for many reasons such as exercise history, exposure to other impacts to the head or body, making it difficult to have true controls matched across all demographical and extraneous factors. Scoping reviews are broad in nature and allow for the development of theoretically connections regardless of quality, which allows for a broader and more contextual overview than a systematic review but may be more subject to individual bias. Rigorous and reproducible methods were employed to demonstrate the authors commitment to publish all findings whether findings were positive, negative or not significant to the theoretical argument presented in the introduction.

## Conclusions

While preliminary work exploring the role the cervicocepahlic network plays in control of both static posture and locomotion demonstrates clear connections between deficit cervical afferent input and erroneous motor output. Moreover, morphological changes to the cervical spine and associated sensorimotor deficits were observed throughout the literature in response to contact sport participation. However, while both deficit cervical afferent and erroneous motor output have been observed in response to contact sport participation little research has explored a potential causal relationship between those deficits and future musculoskeletal injury risk. Further research is necessary to elucidate the relationship between cervical pathology and alterations in cervical sensorimotor function in recreational and amateur athletes.

## Supplementary Information

**Additional file 1.** Search strategy.

## Data Availability

Data sharing is not applicable to this article as no datasets were generated or analyzed during the current study.
