# Supplementary material for: The association between contact sport exposure and cervical sensorimotor dysfunction: a scoping review of implications for future musculoskeletal injury risk
Source: Chiropr Man Therap. 2022 Nov 24;30:50. doi: 10.1186/s12998-022-00458-w (PMC9701076; doi:10.1186/s12998-022-00458-w)
Supplement: Supplementary file 1 — Additional file 1. Search strategy. [file 12998_2022_458_MOESM1_ESM.docx]

**Appendix A**

Searching protocol -electronic databases Scoping Review -Cervical Spine and Contact Sport

1^st^ search was performed to identify connection between contact sport participation and any outcomes involving the cervical spine

The main category- terms for that search were:

1. **‘contact sport’** and/ or **‘cervical spine’** (Both of these terms were used each time in any combination with the following secondary terms. Boolean terms such as AND and OR were used to link the main category terms to the secondary terms and use the term OR between secondary terms)

Secondary terms include:

Range of motion OR ‘musculoskeletal injury’ OR ‘injury risk’ OR sensorimotor OR kinesthesia OR degeneration

Sample searches included:

1.‘contact sport’ AND ‘cervical spine’ AND‘range of motion’ OR ‘musculoskeletal injury’ OR sensorimotor OR degeneration

2. ‘Cervical spine’ AND AND ‘range of motion’ OR ‘musculoskeletal injury’ OR sensorimotor OR degeneration (These search is different from the 1^st^ example as it only includes cervical spine and any of the secondary terms not contact sports)

3 There is no need to do a search that is ‘Contact sport” and secondary octcomes as this article is specific to the cervical spine so we aren’t interested in all the outcomes of contact sports just those related to the cervical spine.

2^nd^ search was to find connection between head and neck position sense or cervical kinesthesia and neuromotor control of the extremities, walking, running etc.

The main category terms for this secondary search were;

**‘Head and neck position sense’** and **‘Cervical kinesthesia’ (**they are esenctially the same thing those so they should be used separately but in combination with the secondary terms)

‘Inury risk’ **OR ‘**musculoskeletal injury’ **OR** coordination **OR** limb **OR** contact sport **OR** neuromotor **OR** posture

**Secondary terms include:**

Sample searches here would look like

1. **‘**Head and neck position sense’ **AND** ‘Inury risk’ **OR ‘**musculoskeletal injury’ **OR** coordination **OR** limb **OR** contact sport **OR** neuromotor **OR** posture
2. ‘Cervical kinesthesia’ **AND** ‘Inury risk’ **OR ‘**musculoskeletal injury’ **OR** coordination **OR** limb **OR** contact sport **OR** neuromotor **OR** posture

Step 1

**PubMed**

1st search: from , all articles, all languages

Search for: ‘Contact sport’ and ‘Cervical Spine’

Hits: 143

2nd search: from 1981, all articles, all languages

Search for: ‘Head and neck position sense’ and ‘Cervical kinesthesia’

Hits: 17

**WEB of Science**

1^st^ search : from 1900, all articles, all languages

Search for: ‘Contact sport’ And ‘Cervical Spine’

Hits: 122, 22 relevant papers

2nd search: from 1900, all articles, all languages

Search for: ‘Head and neck position sense’ and ‘Cervical kinesthesia’

Hits: 10

**Sport Discus**

1st search: from 1900, all articles, all languages

Search for: ‘Contact sport’ And ‘Cervical Spine’

Hits: 8, 3 relevant papers

2nd search: from 1900, all articles, all languages

Search for: “spine injury” and “contact sport” or “athlete”

Hits: 85

**Google Advanced**

1st search: 1900, all articles, all languages

Search for: 'contact sports' and 'cervical spine' or 'musculoskeletal injury' or 'range of motion' or 'injury risk' or 'sensorimotor' or 'kinesthesia' or 'degeneration'

Hits: 330

Once these searches were performed we identified additional articles from the citations of relevant other articles.

**Appendix B**

| Item | Question | Action |
| --- | --- | --- |
| 1 | Did the study discuss cervical spine and contact sport (i.e. soccer, American football, rugby, wrestling, ice hockey participation? | Yes, Move to next question  No, move to question 6 |
| 2 | Is the full manuscript of the article available and published in English? | Yes, Move to the next question  No, Study is excluded |
| 3 | Did the study discuss common acute cervical injuries and or injury rates of cervical spine injuries in contact sport athletes? | Yes, Include study  No, move to question 4 |
| 4 | Did the study discuss common chronic injuries to the cervical spine in contact sport athletes? | Yes, Include study  No, Move to question 5 |
| 5 | Did the study discus long-term health outcomes in contact sport athletes related to the cervical spine? | Yes, Include study  No, Move to question 7 |
| 6 | Did the study discuss the role of cervical sensorimotor function in the neural coordination of the upper and lower extremity | Yes, Include study  No, Move to question 7 |
| 7 | Did the study discuss cervical injury /cervical sensorimotor dysfunction and secondary musculoskeletal injury | Yes, Include study  No, Study is excluded |
